# First report of *Cryptosporidium viatorum* and *Cryptosporidium occultus* in humans in China, and of the unique novel *C. viatorum* subtype XVaA3h

**DOI:** 10.1186/s12879-019-4693-9

**Published:** 2020-01-07

**Authors:** Ning Xu, Hua Liu, Yanyan Jiang, Jianhai Yin, Zhongying Yuan, Yujuan Shen, Jianping Cao

**Affiliations:** 1 Key Laboratory of Parasite and Vector Biology, Ministry of Health, Shanghai, 200025 China; 2 National Institute of Parasitic Diseases, Chinese Center for Disease Control and Prevention, Shanghai, 200025 China; 3 Chinese Center for Tropical Diseases Research, Shanghai, 200025 China; 4 WHO Collaborating Centre for Tropical Diseases, Shanghai, 200025 China; 5 National Center for International Research on Tropical Diseases, Ministry of Science and Technology, Shanghai, 200025 China

**Keywords:** *Cryptosporidium*, Subtype, *C. viatorum*, *C. occultus*, Rural area

## Abstract

**Background:**

*Cryptosporidium* is a genus of common intestinal protozoa, members of which cause diarrhea in a wide variety of hosts. Previous studies on *Cryptosporidium* in China have mainly focused on diarrhea sufferers, children, and immunodeficient individuals such as HIV/AIDS patients. However, the epidemiological characteristics of *Cryptosporidium* in the population in rural areas remain unclear. Herein, we investigated the prevalence of, and risk factors for, *Cryptosporidium* in rural areas of Binyang County, Guangxi Zhuang Autonomous Region, China, and genetically characterized the *Cryptosporidium* isolates we obtained.

**Methods:**

From August to December 2016, two villages in Binyang County, Guangxi, were sampled using a random cluster sampling method. Fresh fecal samples were collected from all eligible residents (residence time > 6 months). Molecular characterization of *Cryptosporidium* was carried out based on its SSU rRNA, gp60, actin and hsp70 gene sequences. Fisher’s exact test were conducted to assess the risk factors for *Cryptosporidium* infection.

**Results:**

A total of 400 fecal samples were collected from 195 males (48.8%) and 205 females (51.2%). Two samples (0.5%) were positive for *Cryptosporidium* and were identified as *C. viatorum* and *C. occultus* respectively. Moreover, a new *C. viatorum* subtype XVaA3h was identified based on the sequence of the gp 60 gene.

**Conclusions:**

To our knowledge, this is the first report of *C. viatorum* and *C. occultus* infections in humans in China and of *C. viatorum* subtype XVaA3h. The findings provide important information on the prevalence of *Cryptosporidium* in the Chinese population, and expand the range of *Cryptosporidium* species known to infect people in China.

## Background

Members of the *Cryptosporidium* genus of intestinal protozoa infect a wide range of hosts including humans, non-human primates, birds, amphibians, fish and reptiles [1]. Global concern was raised following an outbreak of cryptosporidiosis in Wisconsin, USA, in 1993, in which 403,000 individuals were affected and 100 fatalities were reported [2]. Only small quantities of oocysts are needed to establish infection, and once food or drinking water is contaminated with *Cryptosporidium* oocysts, or humans come into contact infected individuals or animals, cryptosporidiosis outbreaks may occur [3]. Cluster outbreaks of cryptosporidiosis reported worldwide have represented a great threat to public health [3, 4]. Symptoms of human cryptosporidiosis range from self-limiting diarrhea in (mainly) immunocompetent individuals, to persistent diarrhea (especially in children younger than 5-years-old). Cryptosporidiosis has been described as the second ranked causative agent of diarrhea in children in developing countries in southern Africa [5]. However, no effective drugs or vaccines have been developed, hence early detection and tracing the source of infection are of great importance for preventing outbreaks of cryptosporidiosis.

Globally, cryptosporidiosis is more endemic in developing countries than in developed countries [6]. In China, the reported human prevalence of *Cryptosporidium* has ranged from 0.0 to 16.5% since the first two cases were reported in Jiangsu Province in 1987, and a strong correlation was found between the infection and HIV/AIDS [7–12]. Moreover, *Cryptosporidium* was responsible for about 1.4 to 10.4% of diarrhea episodes in China [9].

To date, at least 39 species of *Cryptosporidium* have been identified [13–16], and at least 21 species are considered zoonotic, among which *C. hominis* and *C. parvum* are the two main pathogens causing cryptosporidiosis in humans [17]. However, in recent years, with the development of molecular biological technologies, the number of cryptosporidiosis cases confirmed to be caused by other *Cryptosporidium* species has increased, and some species or genotypes are predominant in specific countries or regions. For example, studies in Jiangsu and Shanghai, China, revealed unusually high prevalence of *C. andersoni* in diarrhea patients [17, 18]. *C. cuniculus*, for which the natural host is rabbit, was found in patients with diarrhea in the UK [19], and *C. xiaoi*, mainly found in sheep and goat, was detected in HIV/AIDS sufferers with clinical manifestations including vomiting [20]. To our knowledge, seven species of *Cryptosporidium—C. hominis*, *C. parvum*, *C. andersoni*, *C. meleagridis*, *C. felis*, *C. canis* and *C. suis* have been identified in humans in China [1]*.*

At present, cryptosporidiosis is not included in the National Disease Reporting System in China [21], and *Cryptosporidium* oocyst examination is not performed routinely on patients with diarrhea or other gastrointestinal symptoms, so the prevalence of *Cryptosporidium* in China may be underestimated. The present study aimed to investigate the prevalence, risk factors and species/genotype distribution of *Cryptosporidium* among a rural population in China, which are of great relevance to public health.

## Methods

### Study area

Guangxi is situated in southern China, between 22°54′–26°24′ N and 104°26′–112°04′ E. It borders the Beibu Gulf to the south, and is adjacent to Vietnam in the southwest. Binyang County, which belongs to Nanning City of Guangxi Zhuang Autonomous Region, is located in the south–central part of Guangxi and the northeast part of Nanning City. It is high-altitude in the south and low-altitude in the north, surrounded by earthy mountains to the east, south and west, with an open basin in the middle and a large alluvial plain. It has a subtropical monsoon climate with abundant light and heat, long summer and short winter, and abundant rainfall.

### Study design and population

A cross-sectional survey was carried out to investigate the prevalence of, risk factors for, and species/genotype distribution of *Cryptosporidium* in a rural population in Binyang County. The study was conducted in two villages (A and B) from two towns (village A from town C1, and village B from town C2) selected at random. A total of 400 individuals were involved in our study. All participants were grouped according to gender, age, education level, and so on. Males accounted for 48.8% (195/400) and females 51.2% (205/400) of participants. The average age was 35.7 ± 25.3, ranging from 7 months to 89 years old. Participants with primary school education and below accounted for 56.8% (227/400), followed by junior high school level (36.0%; 144/400), and high school education and above (7.2%; 29/400); 65.3% (261/400) of participants reported raising animals at home. Basic information on all participants is shown in Table 1.

**Table 1:** Basic information on participants and assessment of risk factors for *Cryptosporidium* infection

Variable	No. Examined (%)	No. Positive	*p-value*
Gender			
Male	195 (48.8)	2	0.237
Female	205 (51.2)	0	
Age			
< 5 years (infants)	61(15.3)	1	0.502
5–12 years (children)	76(19.0)	0	
13–19 years (youths)	11(2.7)	0	
20–49 years (adults)	91(22.7)	0	
50 years up (elderly)	161(40.3)	1	
Education level			
Primary and below	227 (56.8)	2	0.590
Junior middle school	144 (36.0)	0	
High school and above	29 (7.2)	0	
Drinking water sources			
Tap water	222 (55.5)	0	0.203
Well or spring water	176 (44.0)	2	
Others	2 (0.5)	0	
Drinking unboiled water			
Yes	159 (39.8)	2	0.157
No	241 (60.2)	0	
Washing hands before meals			
Yes	347 (86.8)	2	1.000
No	53 (13.2)	0	
Eating unwashed vegetables and fruits			
Yes	179 (44.8)	2	0.200
No	221 (55.2)	0	
Animals raising			
Yes	261 (65.3)	2	0.545
No	139(34.7)	0	
Diarrhea			
Yes	10 (2.5)	0	1.000
No	390 (97.5)	2	

### Sample collection and questionnaire

From August to December 2016, 400 fresh fecal samples (>5 g) were collected from villagers in the two selected villages. Samples were taken from the middle of the stool to avoid contamination from soil, animal manure or other human feces, and transported to the laboratory of the local Center for Disease Control within 4 h of collection. Samples were mixed with 2.5% potassium dichromate and stored in a refrigerator at 4 °C. All samples were eventually sent to the laboratory of the National Institute of Parasitic Diseases, Center for Disease Control and Prevention of China. During sampling, villagers involved in the survey were presented with a structured questionnaire to collect data on sociodemographic factors (gender, age, education level), and possible risk factors (drinking and eating habits, hygiene habits, animals raising), as well as common clinical symptoms (diarrhea).

### DNA extraction

Fecal samples (180–220 mg) were washed with deionized water and centrifuged at 20,000 g for 10 min three times to remove potassium dichromate, and DNA was extracted using a QIAamp DNA Stool Mini Kit (Qiagen, Hilden, Germany) following the manufacturer’s instructions. To increase the DNA yield, the lysis temperature was adjusted to 95 °C according to the manufacturer’s recommendation. The final 200 μl DNA samples were stored at − 30 °C for subsequent Polymerase Chain Reaction (PCR) analysis.

### PCR amplification and sequencing

Nested PCR was used to identify *Cryptosporidium* species/genotype, initially by amplifying part of the small-subunit rRNA (SSU rRNA) gene (approximately 840 bp) of *Cryptosporidium* using primer sets and cycling parameters described previously [22]. Primers used to amplify an 805-bp fragment of the 60-kDa glycoprotein-encoding (gp60) gene of *C. viatorum* were described by Stensvold [23]. Heat shock protein 70 (hsp70) and actin genes of *C. occultus* were amplified using primers described by Sulaiman [24, 25] (see Additional file 1: Table S1).

All DNA samples were analyzed at least three times. A *Cryptosporidium*- positive DNA (cattle-derived *C. andersoni*) sample and nuclease-free water were used as positive and negative controls, respectively, and PCR products were analyzed by 2% agarose gel electrophoresis and ethidium bromide staining. Products of the expected size were analyzed using an ABI 3730 DNA Analyzer (Applied Biosystems, Foster City, USA) and Big Dye Terminator v3.1 Cycle Sequencing Kit (Applied Biosystems). ContigExpress software was used for sequence assembly and wave peak evaluation. Sequences were searched using the basic local alignment search tool (BLAST) and aligned with representative *Cryptosporidium* sequences.

### Phylogenetic analysis

Phylogenetic trees were constructed using MEGA6.0 software [26] based on 1000 bootstrap replicates from sequences obtained in this study and representative *Cryptosporidium* sequences downloaded from the NCBI database. The subtype of *C. viatorum* was designated using the subtype naming guidelines [24].

### Statistical analysis

SPSS 16.0 software was used for statistical analyses. Fisher’s exact test were performed to compare infection rates between groups. Differences were regarded as statistically significant when *P* < 0.05.

## Results

### The prevalence of and risk factors for *Cryptosporidium* infection

The prevalence of *Cryptosporidium* was 0.5% (2/400). All 400 participants completed the questionnaire effectively. In the present study, several factors were analyzed. Univariate analysis of risk factors for *Cryptosporidium* are summarized in Table 1. No statistical association was found between these factors and *Cryptosporidium* infection.

Both infected individuals were males from two villages. The person infected with *C. viatorum* was a 60-year-old man with gastric cancer who raised dogs. The other, infected with *C. occultus*, was a child younger than 5 years old who often drank unboiled water, and dogs and chickens were raised in his home (Table 2)*.* Both *Cryptosporidium*-infected individuals failed to display gastrointestinal symptoms.

**Table 2:** Characteristics of the two *Cryptosporidium* infection cases identified in Guangxi, China

Characteristics	Case 1	Case 2
Gender	Male	Male
Age	60	4
Education level	Primary school	Below primary school
Drinking water source	Spring water	Well water
Drinking unboiled water	Yes	Yes
Animals raising	Yes (dogs)	Yes (dogs and chickens)
Contact with animals	Yes	Yes
Diarrhea	No	No
Nutritional status	Poor	General
Other diseases	Yes (gastric cancer)	No
Sampling time	August	December

### Genotype of *Cryptosporidium* spp

Two samples were identified as *Cryptosporidium*-positive, and they corresponded to *C. viatorum* and *C. occultus* respectively, based on the SSU rRNA gene sequences*.* The gp60 gene of *C. viatorum* and actin and hsp70 genes of *C. occultus* were also successfully amplified. A novel subtype of *C. viatorum* was found and named XVaA3h.

*Cryptosporidium* sequences identified in the present study were submitted to GenBank under accession numbers MH807495 (*C. viatorum* SSU rRNA gene), MH807494 (*C. viatorum* gp60 gene), MH807493 (*C. occultus* SSU rRNA gene), MN177696 (*C. occultus* actin gene) and MN177697 (*C. occultus* hsp70 gene),

### Phylogenetic analysis of *C. viatorum* and *C. occultus*

**Fig. 1:**
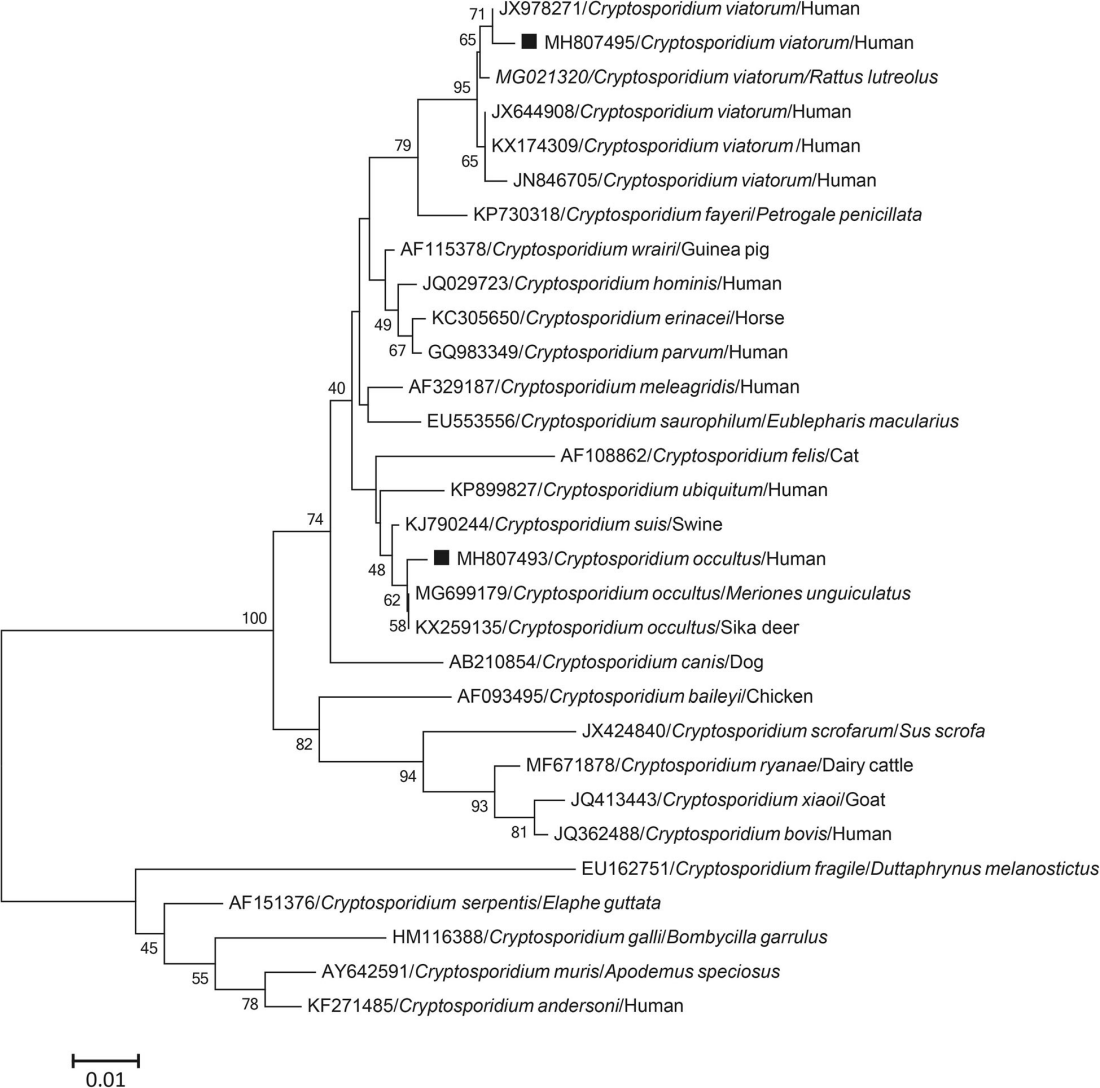
Phylogenetic relationships between *Cryptosporidium* species. Phylogenetic tree based on the partial SSU rRNA gene sequence of *Cryptosporidium* species constructed by the neighbor-joining distance method with 1000 bootstrap replicates using MEGA 6.0 software. Individual GenBank accession numbers precede species, which are followed by host names. Squares represent *Cryptosporidium* sequences from this study

**Fig. 2:**
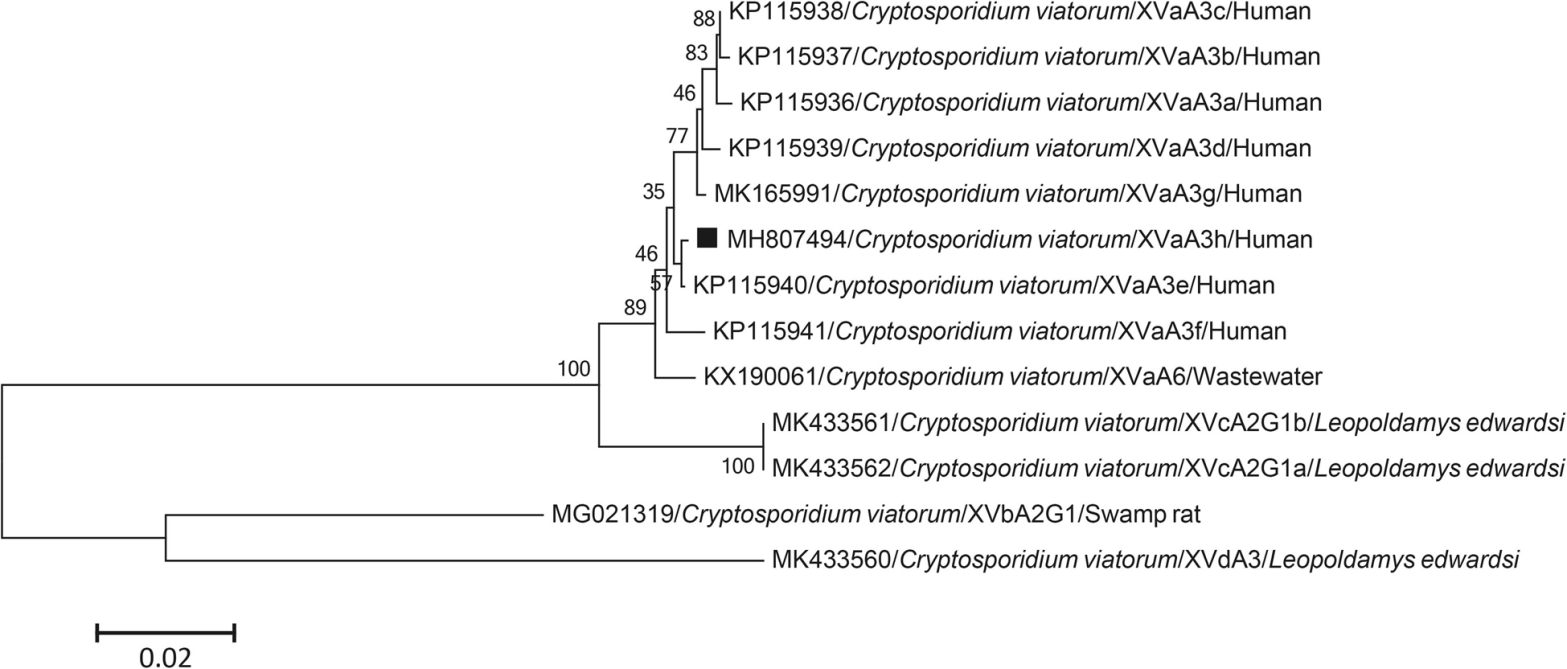
Phylogenetic relationships between different subtypes of *C. viatorum*. Phylogenetic tree based on gp60 gene sequences of *C. viatorum* constructed by the neighbor-joining distance method with 1000 bootstrap replicates using MEGA 6.0 software. Individual GenBank accession numbers precede species and subtype names, which are followed by host names or water. The square indicates the *C. viatorum* subtype identified from a stool DNA sample in this study

Phylogenetic analysis based on SSU rRNA gene sequences confirmed that one sample belonged to *C. viatorum*, and it was closely related to an isolate identified previously in a human in Kenya (accession no. JX978271) (Fig. 1), with one genetic variation. Sequence analysis based on the gp60 gene revealed three contiguous TCA trinucleotides in the serine repeat region, and two genetic variations in the non-repetitive region compared with an isolate from Nepal (accession no. KP115940). The sequence was designated XVaA3h (accession no. MH807494) (Fig. 2).

**Fig. 3:**
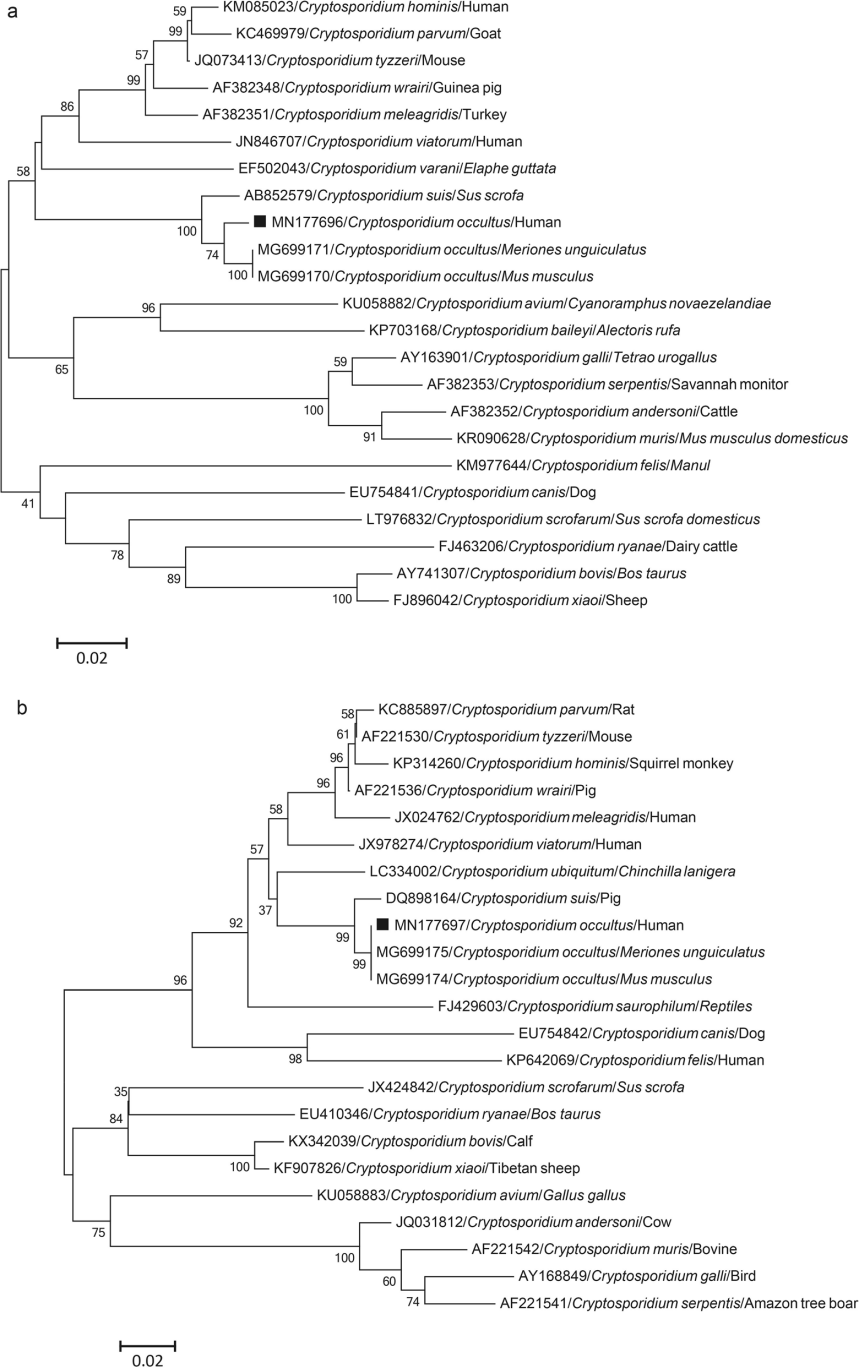
Phylogenetic relationships between different species of *Cryptosporidium* based on actin gene (**a**) and heat shock protein 70 (hsp70) gene (**b**) sequences. The phylogenetic tree was constructed by the neighbor-joining distance method with 1000 bootstrap replicates using MEGA 6.0 software. Individual GenBank accession numbers precede species names, which are followed by host names. The square indicates *C. occultus* identified from a stool DNA sample in this study

The other positive sample clustered with *C. occultus* sequences isolated from deer in China (accession no. KX259135) and *Meriones unguiculatus* in the Czech Republic (accession nos. MG699171 and MG699175) based on SSU rRNA, actin and hsp70 gene sequences, with which it shares > 99% sequence identity (Figs. 1 and 3).

## Discussion

Traditionally, the identification of *Cryptosporidium* mainly depends on microscopy, which is time-consuming and experience-requiring, and this method is not applicable in the case of mild infection [27]. Compared with traditional morphological methods, molecular techniques are more sensitive for detection of *Cryptosporidium* in feces [17]. Nested PCR has been widely used in the detection of *Cryptosporidium.* Molecular epidemiology study on *Cryptosporidium* has been conducted in several provinces/cities/autonomous regions of China, including Guangxi, Guangdong, Henan, Hubei, Jiangsu, Shanghai, Xinjiang and Yunnan, with the prevalence ranging from 0.0 to 16.5% [7, 8, 10–12, 17, 18, 28–30] (Table 3). In the present study, two *Cryptosporidium*-positive samples were detected based on the SSU rRNA gene, revealing a prevalence of 0.5%, which is lower than that reported previously for a rural population in southwest China (12.6%) and diarrhea patients in Jiangsu (9.9%) and Shanghai (13.5%) [17, 18, 31]. Comparing with the prevalence of *Cryptosporidium* in humans in other countries, this is lower than that reported for children in Egypt (1.4%), Cambodia (7.7%), and rural Ghana (5.2%) and diarrhea patients in Canada (15.7%) [32–35], but higher than that reported in a population-based laboratory surveillance in a large Canadian health region (~ 6.0 per 100,000 members of the population per year) [36]. The prevalence of *Cryptosporidium* varies from country to country, and even within different areas in the same region, which may be related to the immune status, living environment, and even sampling time of the selected subjects.

**Table 3:** Species, positive rate, and risk factors associated with *Cryptosporidium* infection in humans in China

Location	Positive no./Examined no. (%)	Species (no.)	Population	Risk factor	References
Guangxi	6/258 (2.3)	*C. andersoni* (4) *C. hominis* (2)	HIV-positive	No analysis	[28]
Guangxi	2/285 (0.7) 0/150	*C. andersoni* (1), *C. hominis* (1)	HIV-positive HIV-negative	HIV-positive with diarrhea Location (Guilin)	[8]
Guangdong	12/348 (3.5)	*C. hominis* (8) *C. parvum* (4)	Diarrheal children	No risk factor	[29]
Henan	10/673 (1.5) 1/628 (0.2)	*C. meleagridis* (5), *C. hominis* (3) *C. parvum* (2) *C. suis* (1)	HIV-positive HIV-negative	HIV infection, Raising sheep/goat Well water as water source	[10]
Hubei	9/298 (3.0)	*C. parvum* (9) *C. hominis* (2) two with mixed species	Diarrheal infants <2 years old	Children with diarrhea aged 1–2	[7]
Jiangsu	23/232 (9.9)	*C. andersoni* (21)*, C. hominis* (2),	Diarrheal outpatients	Autumn	[17]
Shanghai	34/252 (13.5)	*C. andersoni* (34*)*	Diarrheal outpatients	Winter	[18]
Shanghai	102/6284 (1.6)	*C. hominis* (92) *C. meleagridis* (6) *C. canis* (2) *C. felis* (2)	Children in hospitals	Children < 6 months, February–July 2008 Diarrhea	[11]
Xinjiang	38/230 (16.5)	*C. hominis* (−) *C. parvum* (−)	Diarrheal patients	No analysis	[12]
Yunnan	1/850 (0.1) 0/170	–	Asymptomatic and diarrheic children	No risk factor	[30]

Note: These data are based on molecular methods

*Cryptosporidium* is a common opportunistic pathogen in immunodeficient/immunocompromised individuals, especially HIV-infected patients, cancer patients and children younger than 5 years old [10, 29, 37]. In the present study, one case was a cancer patient and another was a child younger than 5 years old, both of whom were susceptible to *Cryptosporidium*. Age is one of the most important factors for cryptosporidiosis [7, 11], but no statistical association was observed in our present study, which could be mostly attributed to the population groups. Indeed, a previous study conducted in an underdeveloped rural community of southwest China found no association between infection and age groups [38] .

Diarrhea is the most common clinical symptom associated with *Cryptosporidium* infection, and a statistically significant association between *Cryptosporidium* infection and diarrhea has been reported previously [39]. However, no statistical association was observed between *Cryptosporidium* infection and diarrhea in the present study (*p* = 1.000) which could be attributed to the species or subtypes of *Cryptosporidium*. The relationship between diarrhea and these two *Cryptosporidium* species (*C. viatorum* and *C. occultus*) remains unclear due to the lack of cases.

*Cryptosporidium* is a waterborne parasite and listed as one of the indispensable indicators of water quality according to the Hygienic Standards for Drinking Water in China (GB 5479–2006). However, in this study, statistically significant difference was not observed between individuals drinking unboiled water or not, which is contrary to the results of a study conducted in Russia [40]. The reason may be that people drinking boiled water in the present study accounted for 60.2% (241/400) of all the respondents. Although *Cryptosporidium* oocysts are resistant to low temperature and chlorine disinfectant in the external environment, heating at 65–70 °C for 30 min can kill *Cryptosporidium* oocysts. Some other factors (e.g. gender, education level, drinking water source, washing hands before meals, eating unwashed vegetables and fruits, and raising animals) were also analyzed, but no statistical correlation was found between those factors and *Cryptosporidium* infection.

Globally, *C. hominis* and *C. parvum* are the most common species causing cryptosporidiosis in humans, accounting for > 90% of cases [41]. Seven species of *Cryptosporidium* have been identified in humans in China with *C. hominis* and *C. parvum* being the commonest, but the distribution of *Cryptosporidium* species in China varies by region; *C. hominis* is mainly present in the east, while *C. parvum* is mainly found in the south–central region (Hunan Province) [42–44]. In the present study, *C. viatorum* and *C. occultus* were identified for the first time in humans in China.

*C. viatorum*, previously believed to be a human-specific pathogen, was first isolated from travelers returning to Britain from India in 2012 [45]*. C. viatorum* has since been identified in people from or who travelled to Bangladesh, Barbados, Colombia, Dubai, Ethiopia, Guatemala, India, Kenya, Nepal, Nigeria, Pakistan and Australia [20, 23, 45–53]. Recently, researchers in Australia and China identified *C. viatorum* in rats, underlining its zoonotic potential [54, 55]*.* It was also identified in untreated water in China [56]. In the present study, we identified the subtype XVaA3h of *C. viatorum* in a human for the first time (Table 4).

**Table 4:** *C. viatorum* identified in humans, animals, and water samples worldwide

Country	Host	Year	Travel history	Subtype	References
UK	Human	2012 2015	Yes Bangladesh (1) India (9) Nepal (1) Pakistan (1) Unknown (2)	XVaA3a (9) XVaA3d (2) XVaA3e (1) XVaA3f (2)	[23, 45]
Sweden	Human	2013	Yes Kenya (2) Guatemala (1)	XVaA3b (1) XVaA3c (1) XVaA3d (1)	[23, 46, 47]
Ethiopia	Human	2014 2015 2016	Unknown (22)	XVaA3d (9) Unknown (13)	[20, 23, 48]
Nigeria	Human	2014 2017	Unknown (2)	Unknown (2)	[49, 50]
Colombia	Human	2017	Unknown (1)	Unknown (1)	[51]
China	Waste water	2017	–	XVaA6	[56]
India	Human	2018	Unknown (1)	Unknown (1)	[52]
Australia	Rat	2018	–	XVbA2G1 (1) Unknown (2)	[54]
Australia	Human	2019	Unknown (1)	XVaA3g (1)	[53]
China	*Leopoldamys edwardsi*	2019	–	XVcA2G1a (4) XVcA2G1b (1) XVdA3 (1)	[55]
China	Human	2019	No (1)	XVaA3h (1)	This study

Based on the gp60 gene sequence, which is applied in subtype analysis of several pathogenic *Cryptosporidium* species, including *C. hominis*, *C. parvum*, *C. ubiquitum*, *C. meleagridis*, *C. viatorum*, *Cryptosporidium* skunk genotype and *Cryptosporidium* chipmunk genotype I [56], *C. viatorum* has evolved into 13 subtypes, named XVaA3a–h, XVaA6, XVbA2G1, XVcA2G1a, XVcA2G1b and XVdA3. The sequence of the gp60 gene of XVaA3h identified in our study shares > 98% identity with other *C. viatorum* subtypes, isolated from humans in Nepal (accession no. KP115940), Guatemala (accession no. KP115938), India (accession nos. KP115941 and KP115936) and Kenya (accession no. KP115939), and is 97% identical to an isolate from waste water in China (accession no. KX190061). High genetic identity among *C. viatorum* subtypes XVaA3a–h may suggest that *C. viatorum* recently spread from the source population and is now spreading further through global human travel [54]. However, no travel history was reported in our study. Perhaps animals or contaminated water contribute to the infection, considering the fact that *C. viatorum* has been identified in animals and water [54–56].

*C. occultus*, named by Martin Kvác in 2018 [15], and was previously known as *Cryptosporidium suis*-like, due to the close phylogenetic relationship with *C. suis*. However, oocysts of *C. occultus* are morphologically indistinguishable from other species/genotypes, while oocysts of *C. occultus* are smaller than those of *C. suis* [15]. Previous phylogenetic analyses based on SSU rRNA, actin, and hsp70 genes revealed 0.3, 2.0, and 2.1% sequence divergence from *C. suis*, respectively, hence *C. occultus* and *C. suis* can be distinguished genetically [15].

Although *C. occultus* has a wide host range (cattle, yak, water buffalo, and rat), one study showed that rats are the main host [15]. To date, only two cases of humans infected with *C. occultus* have been reported, in Canada and in England [57, 58]. *C. occultus* has been detected in cattle, yak, and wild rats in China [55, 59, 60]. Our present study is the first report of *C. occultus* in a human in China. However, the transmission route is not clear due to limited detection of *C. occultus* in animals and humans.

Various zoonotic *Cryptosporidium* species (*C. hominis*, *C. parvum*, *C. meleagridis*, *C. andersoni*, *C. felis*, *C. ubiquitum*, *Cryptosporidium* horse genotype, *C. suis* and *C. viatorum*) have been found in different water bodies that may be contaminated by animal and human feces, indicating a transmission cycle of *Cryptosporidium* among humans, animals and water [1, 61]. Study also suggested that water may be contaminated with *C. occultus* [15]. To better understand the transmission dynamics of cryptosporidiosis and provide targeted preventive measures, further molecular investigations of *Cryptosporidium* in animal and water samples are required.

## Conclusions

The present study identified *C. viatorum* and *C. occultus* in humans in China for the first time, and also documents the novel *C. viatorum* subtype XVaA3h, expanding the known range of *Cryptosporidium* species infecting humans worldwide. Those two *Cryptosporidium* species have both been identified in animals, suggesting the possibility of zoonotic transmission of *Cryptosporidium* in this locale. Further systematic molecular investigation of *Cryptosporidium* should focus on humans, animals and water samples to clarify the transmission routes.

## Supplementary information

**Additional file 1: Table S1.** Primers used in the study.

## Data Availability

The datasets generated and/or analysed during the current study are not publicly available in order to protect participant confidentiality. The gene sequences from *Cryptosporidium* identified in this study were submitted to GenBank with accession numbers MH807493–MH807495 and MN177696–MN177697.

## Abbreviations

HIV/AIDS: Human Immunodeficiency Virus/Acquired Immune Deficiency Syndrome
SSU rRNA: small subunit RNA
gp60: 60-kDa glycoprotein-encoding
hsp70: heat shock protein 70
