# Supplementary material for: First report of Cryptosporidium viatorum and Cryptosporidium occultus in humans in China, and of the unique novel C. viatorum subtype XVaA3h
Source: BMC Infect Dis. 2020 Jan 7;20:16. doi: 10.1186/s12879-019-4693-9 (PMC6947842; doi:10.1186/s12879-019-4693-9)
Supplement: Supplementary file 1 — Additional file 1: Table S1. Primers used in the study. [file 12879_2019_4693_MOESM1_ESM.doc]

**Additional t**able 1 Primers used in the study

| Genes | Sequences | Size (bp) | References |
| --- | --- | --- | --- |
| SSU rRNA | F1:TTCTAGAGCTAATACATGCG | ~1320 | [22] |
|  | R1:CCCATTTCCTTCGAAACAGGA |  |  |
|  | F2:GGAAGGGTTGTATTTATTAGATAAAG | ~840 |  |
|  | R2:CTCATAAGGTGCTGAAGGAGTA |  |  |
| gp60 | F1:TTCATTCTGACCCCTTCATAG | ~918 | [23] |
|  | R1:CAAAAACAG AAGGGATGATGTATC |  |  |
|  | F2:GAGATT GTCACTCATCATCGTAC | ~805 |  |
|  | R2:GGAAGAAGAACT TGCATCC |  |  |
| hsp70 | F1:ATGTCTGAAGGTCCAGCTATTGGTATTGA | ~2015 | [24] |
|  | R1:TTAGTCGACCTCTTCAACAGTTGG |  |  |
|  | F2:TATTCATGTGTTGGTGTATGGAGAAA | ~1950 |  |
|  | R2:CAACAGTTGGACCATTAGATCC |  |  |
| actin | F1:ATG(A/G)G(A/T)GAAGAAG(A/T)A(A/G)(C/T)(A/T)CAAGC | ~1095 | [25] |
|  | R1:AGAA(G/A)CA(C/T)TTTCTGTG(T/G)ACAAT |  |  |
|  | F2:CAAGC(A/T)TT(G/A)GTTGTTGA(T/C)AA | ~1066 |  |
|  | R2:TTTCTGTG(T/G)ACAAT(A/T)(G/C)(A/T)TGG |  |  |
